# Transcriptome Analysis and SNP Identification Reveal That Heterologous Overexpression of Two Uncharacterized Genes Enhances the Tolerance of Magnaporthe oryzae to Manganese Toxicity

**DOI:** 10.1128/spectrum.02605-21

**Published:** 2022-05-31

**Authors:** Yi Wang, Lina Liu, Xin Pu, Chan Ma, Hao Qu, Mian Wei, Ke Zhang, Qi Wu, Chengyun Li

**Affiliations:** a State Key Laboratory for Conservation and Utilization of Bio-Resources in Yunnan, Yunnan Agricultural University, Kunming, People's Republic of China; University of Michigan

**Keywords:** *Magnaporthe oryzae*, manganese toxicity, SNP, overexpression

## Abstract

Manganese is a crucial trace element that constitutes the cofactors of many enzymes. However, excessive Mn^2+^ can be toxic for both prokaryotes and eukaryotes. The mechanism of fungal genetics and metabolism in response to Mn^2+^ stress remains understudied, warranting further studies. Magnaporthe oryzae is well-established as the most destructive pathogen of rice. A field strain, YN2046, more sensitive to Mn^2+^ toxicity than other strains, was obtained from a previous study. Herein, we explored the genetic mechanisms of Mn^2+^ sensitivity in YN2046 through comparative transcriptomic analyses. We found that many genes previously reported to participate in Mn^2+^ stress were not regulated in YN2046. These non-responsive genes might cause Mn^2+^ sensitivity in YN2046. Weight gene correlation network analysis (WGCNA) was performed to characterize the expression profile in YN2046. Some overexpressed genes were only found in the Mn^2+^ tolerant isolate YN125. Among these, many single nucleotide polymorphism (SNP) were identified between YN125 and YN2046, which might disrupt the expression levels of Mn responsive genes. We cloned two uncharacterized genes, *MGG_13347* and *MGG_16609*, from YN125 and transformed them to YN2046 with a strong promoter. Our results showed that the heterologous overexpression of two genes in YN2046 restored its sensitivity. Transcriptomic and biochemical analyses were performed to understand Mn tolerance mechanisms mediated by the two heterologous overexpressed genes. Our results showed that heterologous overexpression of these two genes activated downstream gene expression and metabolite production to restore M. oryzae sensitivity to Mn, implying that SNPs in responsive genes account for different phenotypes of the two strains under Mn stress.

**IMPORTANCE** Heavy metals are used for fungicides as they target phytopathogen in multiple ways. Magnaporthe oryzae is the most destructive rice pathogen and is threatening global rice production. In the eukaryotes, the regulation mechanisms of Mn homeostasis often focus on the posttranslation, there were a few results about regulation at transcript level. The comparative transcriptome analysis showed that fewer genes were regulated in the Mn-sensitive strain. WGCNA and SNP analyses found that mutations in promoter and coding sequence regions might disrupt the expression of genes involved in Mn detoxification in the sensitive strain. We transferred two unannotated genes that were cloned from the Mn-tolerant strain into a sensitive strain with strong promoters, and the transformants exhibited an enhanced tolerance to Mn^2+^ toxicity. Transcriptome and biochemistry results indicated that heterologous overexpression of the two genes enhanced the tolerance to Mn toxicity by reactivation of downstream genes in M. oryzae.

## INTRODUCTION

Magnaporthe oryzae is the most destructive plant pathogen and a significant threat to global rice production. In recent years, the infection of wheat by the M. oryzae lineage has caused colossal losses in wheat yields in some American and Asian countries, and M. oryzae is a potential risk to other crops (1). With an increasing number of sequenced M. oryzae genomes (2–4), the genetic differentiation and pathogenic mechanism of M. oryzae have gradually been uncovered, providing a foothold for managing the disease. Moreover, M. oryzae is often regarded as an indispensable model fungus that can be harnessed to improve the current knowledge of fungal molecular biology.

Manganese (Mn) is an essential trace element that is necessary for the production of cofactors, which are involved in enzyme activities such as carbohydrate and nucleic acid metabolism, immunity response, and oxidative stress resistance (5–9). Recent evidence suggests that Mn participates in photosynthesis, and a lack of Mn can cause interveinal chlorosis in young leaves (10, 11). Mn capture is a bacterial approach to resist the oxidative stress that is generated by hosts, and this strategy aids the bacteria in successfully infecting the host (12). Thus, hosts often compete for Mn with pathogens by chelating proteins and importing transporters to suppress bacterial growth (13, 14). Many transporters that are associated with Mn uptake have been identified, and mutants of these transporters can lead to defects in bacterial infection and host immunity (15, 16).

Mn is a component of maneb that is often used for managing plant diseases, and Mn additives in fuel production have been reported to increase the amount of environmental Mn. Moreover, Mn overexposure could facilitate manganism and neurodegenerative disorders (6, 17, 18). For example, Mn toxicity-induced ɑ-synuclein (ɑ-Syn) aggregation has been implicated in Parkinson's disease (PD) (19). Moreover, excess Mn^2+^ can decrease the expression of oxidative phosphorylation-related genes and disrupt complex activities in the electron transport chain, causing mitochondrial dysfunction and an impairment in the generation of cellular energy (20, 21). An overexposure of Mn^2+^ also disrupts metalloprotein activity and inhibits heme and cytochrome oxidase activity through ferrochelatase inactivation (22, 23).

It has been documented that in prokaryotic and eukaryotic organisms, many of the transporters responsible for the movement of Mn also maintain Mn homeostasis. In bacteria, transcription factors and a riboswitch that can detect intracellular Mn levels have been reported to regulate the expression of downstream transporter genes (24). However, eukaryotes such as yeast can reportedly mediate the posttranslation of Mn homeostasis to degrade Mn-related proteins and alter cellular Mn levels (25). Thus, it is essential to explore novel Mn regulation pathways at the transcriptional level in eukaryotic organisms. Moreover, many Mn complexes that are formed with orthophosphate, carbonates, peptides, nucleosides, and organic acids that are associated with oxidative stress resistance have been found in prokaryotes and yeast (8). Intriguingly, *Lactobaccilus plantarum* can tolerate 20 mM Mn metabolites to mimic superoxide dismutase (SOD), and this is a higher Mn content than that tolerated by other organisms (26), suggesting many microorganisms exhibit strong abilities to biologically remove Mn (27, 28). Therefore, the formation of Mn complexes also plays an essential role in Mn detoxification and oxidative stress resistance.

M. oryzae is a model filamentous fungus that has attracted much interest due to its ability to undergo easily genetic manipulation, abundant genomic references, and economic value. Uncovering the molecular biology of M. oryzae provides management strategies for rice blast disease and establishes the regulatory networks regarding the preferred traits of plant pathogenic fungi. In a previous study, we identified an Mn^2+^-associated gene, *MoMCP1*, which confers resistance to toxic Mn^2+^ and is involved in pathogenicity in M. oryzae (20). Moreover, in a previous study, we reported a novel field strain, YN2046, which had a greater sensitive to Mn^2+^ toxicity than that of other isolates and provided valuable material for studying Mn^2+^ toxicology in rice blast fungus. Herein, we characterized the gene expression pattern in YN2046 under Mn^2+^ toxicity. The results showed that many genes that were previously reported to be involved in Mn^2+^ detoxification were not expressed in YN2046. According to weighted gene correlation network analysis (WGCNA) and single nucleotide polymorphism (SNP) analyses, we cloned two genes from an Mn-tolerant rice blast fungus, YN125, and transferred them with a strong promoter into YN2046. The overexpression of *MGG_13347* and *MGG_16609* exhibited tolerance to Mn^2+^ toxicity, which restored the expression of downstream genes, enzyme activities, and metabolite levels.

## RESULTS

### Among the rice blast strains, YN2046 was more sensitive to excessive Mn^2+^.

**FIG 1:**
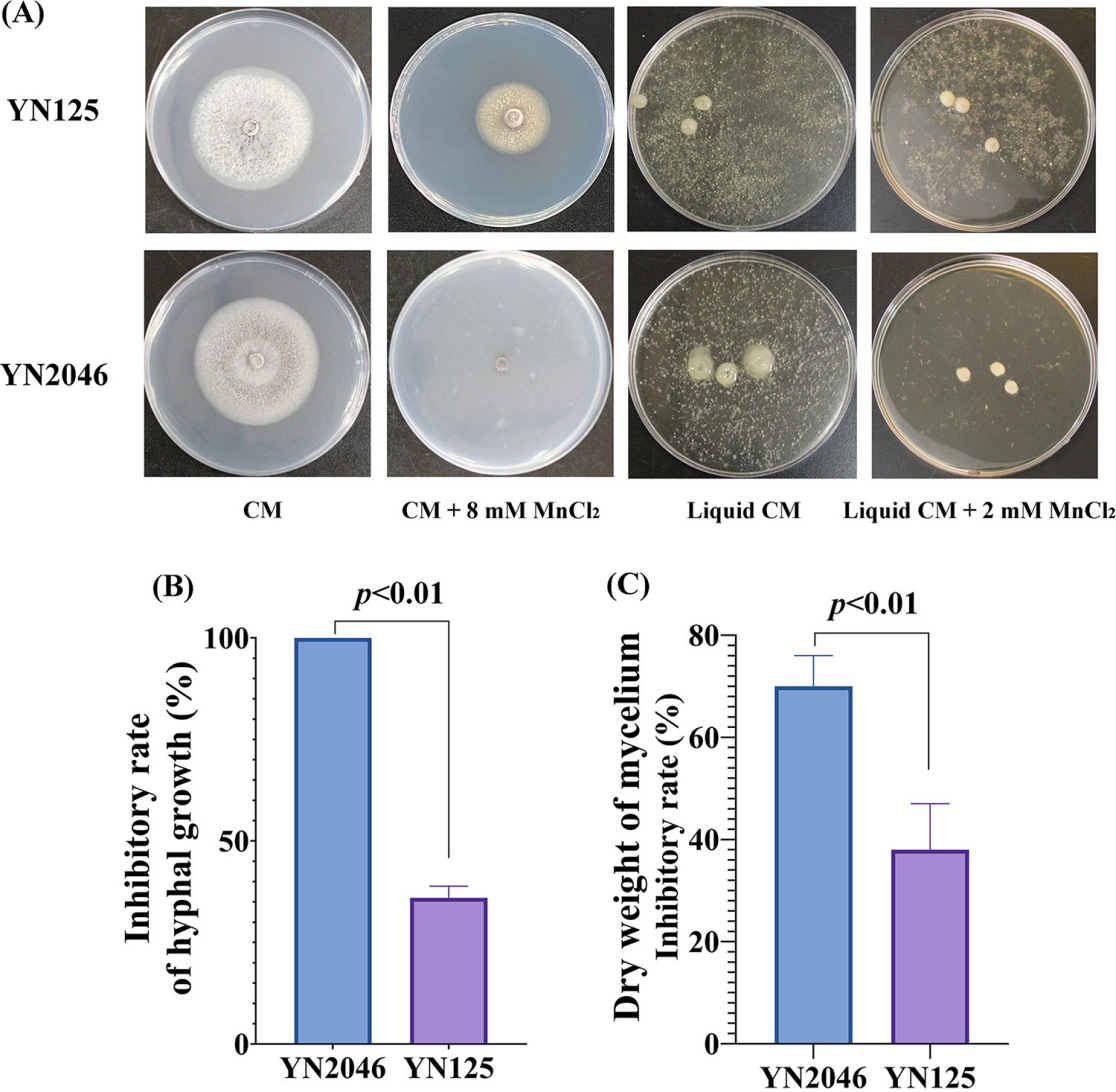
The M. oryzae strain YN2046 was more sensitive to excessive Mn^2+^. (A) The colonies of YN2046 and YN125 were cultivated in complete solid medium with 8 mM MnCl_2_ and complete liquid medium with 2 mM MnCl_2_. The inhibitory rates of hyphal growth (B) and mycelium dry weight (C) with Mn treatment. The results were obtained from three independent experiments. Statistically significant differences were calculated by Student's *t* test. Error bars represent the means ±SE.

We previously established that the inhibitory effects of excessive Mn^2+^ on different M. oryzae strains collected from the fields were heterogeneous (unpublished data), suggesting that there is a complicated genetic background and sophisticated regulatory network in response to excess Mn^2+^ in M. oryzae. In this study, among the strains, the M. oryzae strain YN2046 was more sensitive to excess Mn^2+^ (Fig. 1A). The hyphal growth and dry weight in YN2046 exhibited significantly higher inhibitory rates than those in YN125 (Fig. 1B and C), indicating that YN2046 has significant value for research involving Mn^2+^ toxicity. Accordingly, we performed transcriptome analyses to reveal the gene expression signatures in the Mn^2+^-sensitive strain YN2046.

### Gene expression pattern in YN2046 under excess Mn^2+^.

**FIG 2:**
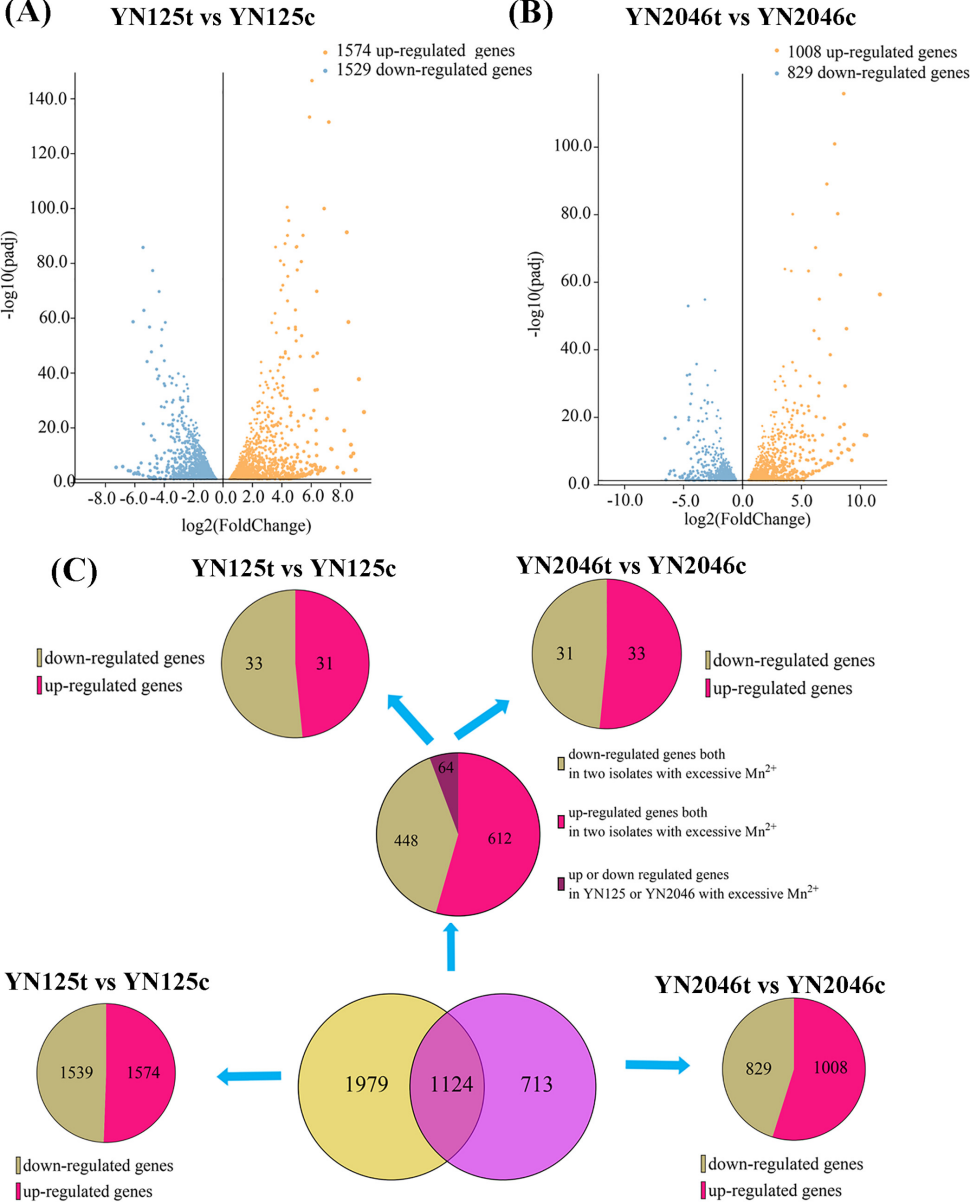
Comparison of differentially expressed genes between YN2046 and YN125 under excess Mn^2+^ treatment. Volcano plots (A and B) and Venn plots (C) show the transcriptomic features between YN2046 and YN125 under excess Mn^2+^ treatment. “t” indicates the strains treated with Mn, and “c” indicates the strains under normal conditions.

To reveal the specific gene expression pattern that was associated with Mn^2+^ stress in YN2046, previously reported Mn^2+^-associated transcriptomic data for YN125 were used as a reference (20). A total of 1,008 upregulated and 829 downregulated genes were significantly regulated in YN2046 (Table S1), and the number of differentially expressed genes (DEGs) in YN2046 was smaller than that in YN125 (1,574 upregulated and 1,529 downregulated genes) (Fig. 2A and B). The intersection of DEGs in YN2046 and YN125 yielded 1,124 DEGs (1,060 genes exhibited identical expression levels, while the expression of 64 genes was downregulated or upregulated in YN2046 or YN125). For strain-specific DEGs, 713 and 1,979 unique genes were regulated in YN2046 and YN125 under excess Mn^2+^, respectively (Fig. 2C). Interestingly, genes that were previously associated with the alleviation of Mn toxicity were not expressed in YN2046 (Fig. S1). Our results indicated that differences in the number of DEGs and gene expression levels under Mn^2+^ stress accounted for heterogeneous phenotypes in response to Mn^2+^ stress between YN2046 and YN125. These findings suggested the activated genes that occur in response to excessive Mn^2+^ were lacking.

Kyoto Encyclopedia of Genes and Genomes (KEGG) pathway and Gene Ontology (GO) enrichment analyses were used to clarify the functions of strain-specific DEGs. Compared with previously reported Mn^2+^-associated transcriptome results in YN125, novel enrichment gene sets that were regulated by excess Mn^2+^ were identified in YN2046. Upregulated genes were significantly enriched in GO terms, including mycelium development, integral component of membrane, phosphopantetheine binding, and transferase activity (Fig. S2A, Table S2), while downregulated genes were significantly enriched in the GO terms associated with DNA and RNA processes (Fig. S2B, Table S2). During KEGG pathway analysis, downregulated DEGs were enriched in the cell cycle, in protein processing in the endoplasmic reticulum, in meiosis and in DNA replication, while upregulated DEGs were involved in DNA repair-related pathways (Fig. S3, Table S3), implying that an excess of Mn^2+^ could decrease fungal proliferation by disrupting DNA synthesis and the cell cycle.

### Weighted gene correlation network analysis.

**FIG 3:**
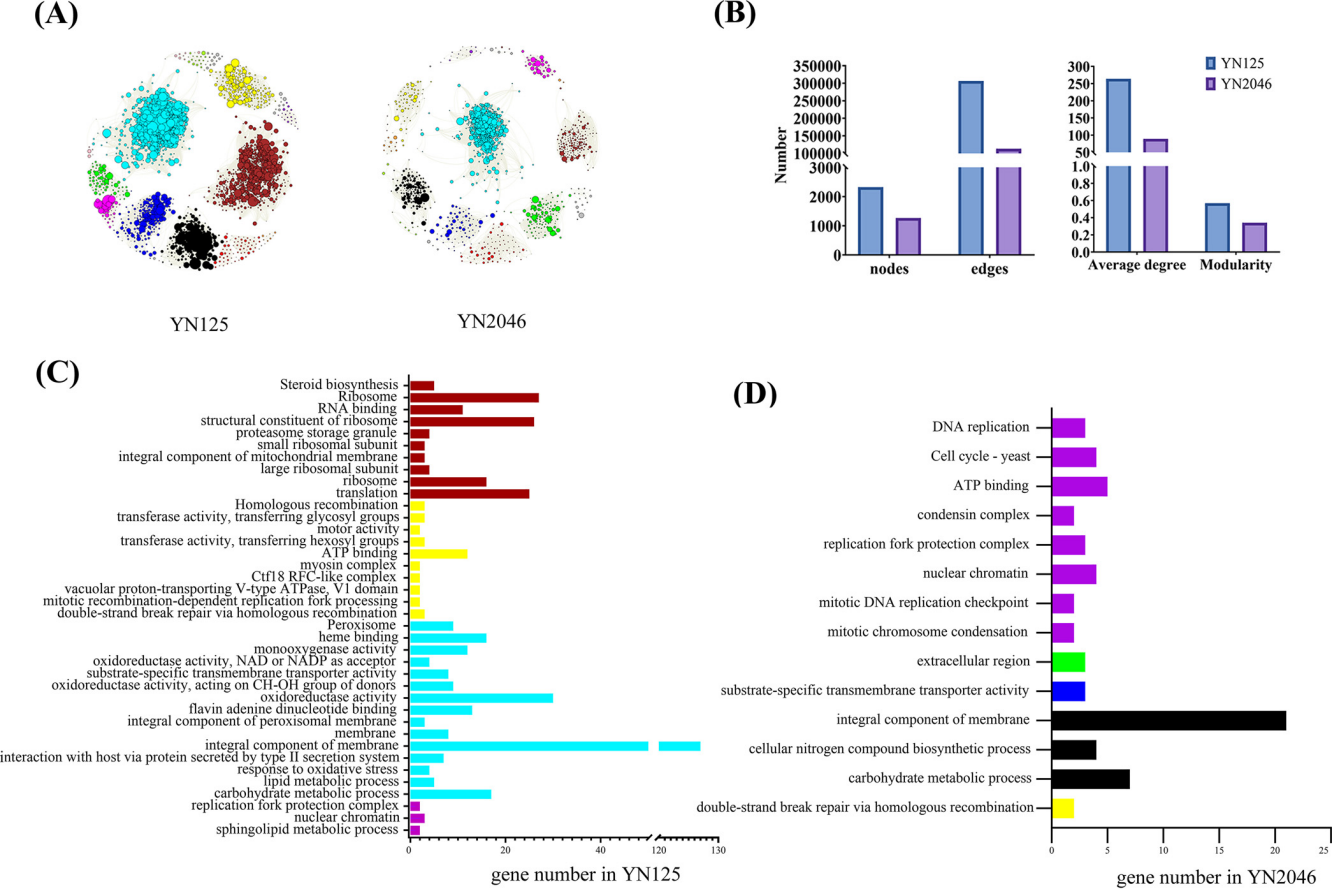
The interaction network and functional enrichment analyses of different modules in YN125 and YN2046. (A) The interaction networks of DEGs among different modules in YN125 (left) and YN2046 (right). (B) The network parameters include nodes, edges, average degrees, and modularity in YN125 and YN2046. Functional enrichment analyses of DEGs in different modules with a significant relationship in YN125 (C) and YN2046 (D).

WGCNA is a valuable tool for analyzing the characteristics of various gene expression clusters and interactions between preferred traits and gene expression patterns. To better understand the candidate genes that regulate Mn^2+^ sensitivity in YN2046, we reviewed the literature for published Mn^2+^ transcriptome profiles in M. oryzae, and a total of 18 samples were used for WGCNA (20) (Table S4). We found that the three biological replicates of each treatment were clustered together, indicating that our samples have a high repeatability (Fig. S4). A power value of β = 20 (R^2^ = 0.81, mean connectivity = 18.40, Fig. S5, Table S5) was selected, and 13 modules were obtained (Fig. 3A, Table S6). The TOM plot was used to analyze associations among DEGs (Fig. S6A and B). We found that the turquoise, brown, blue, and black modules contained more DEGs in YN125. Similarly, the number of DEGs in the turquoise, brown, black, and green modules was higher in YN2046. There were 2,324 nodes and 306,485 edges in YN125, and 1,266 nodes and 112,949 edges were found in YN2046. Moreover, a lower average degree and worse modularity were found in YN2046 (Fig. 3B). These results suggested that looser connections among DEGs might be sensitive to Mn toxicity in YN2046.

The analysis results for module-trait relationships between different samples and gene clusters are shown in Fig. S6C. The results demonstrated that transcripts associated with sensitivity to excess Mn^2+^ in YN2046 showed a strong association (*P < *0.05) with blue, black, yellow, green, magenta, and purple modules, while brown, yellow, turquoise, and purple modules were significantly related to transcripts in YN125 under Mn^2+^ treatment. Moreover, we conducted functional enrichment analysis of significantly related modules in YN125 and YN2046 (Fig. 3C and D). Although more modules were significantly associated with YN2046 with excessive Mn treatment, only 14 functional terms were obtained in YN2046 in contrast to the 37 functional terms in YN125, indicating that fewer enrichment terms were associated with fewer DEGs in YN2046. The traits annotated as cell cycle - yeast, cellular nitrogen compound biosynthetic process, condensin complex, DNA replication, extracellular region, mitotic chromosome condensation, and mitotic DNA replication checkpoint were significantly enriched in YN2046, and most were involved in DNA replication, implying that Mn toxicity could disrupt DNA replication and the cell cycle, consistent with the KEGG pathway analysis results.

### SNP analyses and heterologous overexpression of allele genes in YN2046 enhance Mn tolerance and pathogenicity.

The turquoise and brown modules in YN125 exhibited a unique response to excessive Mn, implying that the genes in these two modules favored tolerance. We aligned the sequences of the promoter and open reading frame (ORF) regions of six genes with highly upregulated expression among these two modules in YN125 but not in YN2046 under Mn treatment. The SNPs of *MGG_ 13347* and *MGG_07812* were found in the promoter region, while the ORFs of *MGG_16609* and *MGG_09416* also possessed SNPs with nonsynonymous substitutions (Table 1). These mutant loci in promoter regions might not activate the expression of regulated genes, while SNPs in the ORF regions of YN2046 could generate nonfunctional proteins to regulate downstream genes.

**TABLE 1:** The SNP analyses of candidate genes between YN125 and YN2046^*a*^

Module	Gene ID	Locus_tag	Foldchange		Description	Strains	Loci									
			YN125	YN2046												
Brown	2676137	*MGG_05483*	2.01	None	hypothetical protein	YN125	None									
						YN2046										
	5050626	*MGG_11467*	1.9	None	hypothetical protein	YN125	None									
						YN2046										
							−314	−8	−9							
	5050279	*MGG_13347*	1.26	None	hypothetical protein	YN125	T									
						YN2046		C	C							
Turqiose							25	147								
	12986998	*MGG_16609*	6.81	None	hypothetical protein	YN125	*C (Pro)*	*C (Ser)*								
						YN2046	*T (Thr)*	*A (Pro)*								
							−980	−970	−935	−857	−779	−702	−381	−304	−267	−18
	2683739	*MGG_07812*	6.33	None	hypothetical protein	YN125	G	A	A	G	C	T	C	C	C	G
						YN2046	A	G	G	A	T	C	T	T	T	A
	2680420	*MGG_09416*	6.18	None	hypothetical protein		129	165	186	264	331	335	336	340	342	349
						YN125	G	G	T	G	G	G	C	C		A
						YN2046	A	A		A	A	A	T	A	A	C
							354	363	372	375	446	449	512	614	670	682
						YN125	A	T	T	G	G	A	G	C		A
						YN2046	C		C	A	C	G	T	T	T	G
							923	975	987	1001	1014	1017	1508			
						YN125	C	T	G		T	G	*A (Asn)*			
						YN2046		G	C	T	C	T	*G (Gly)*			

a The italic letter means nonsynonymous substitution between two strains.

**FIG 4:**
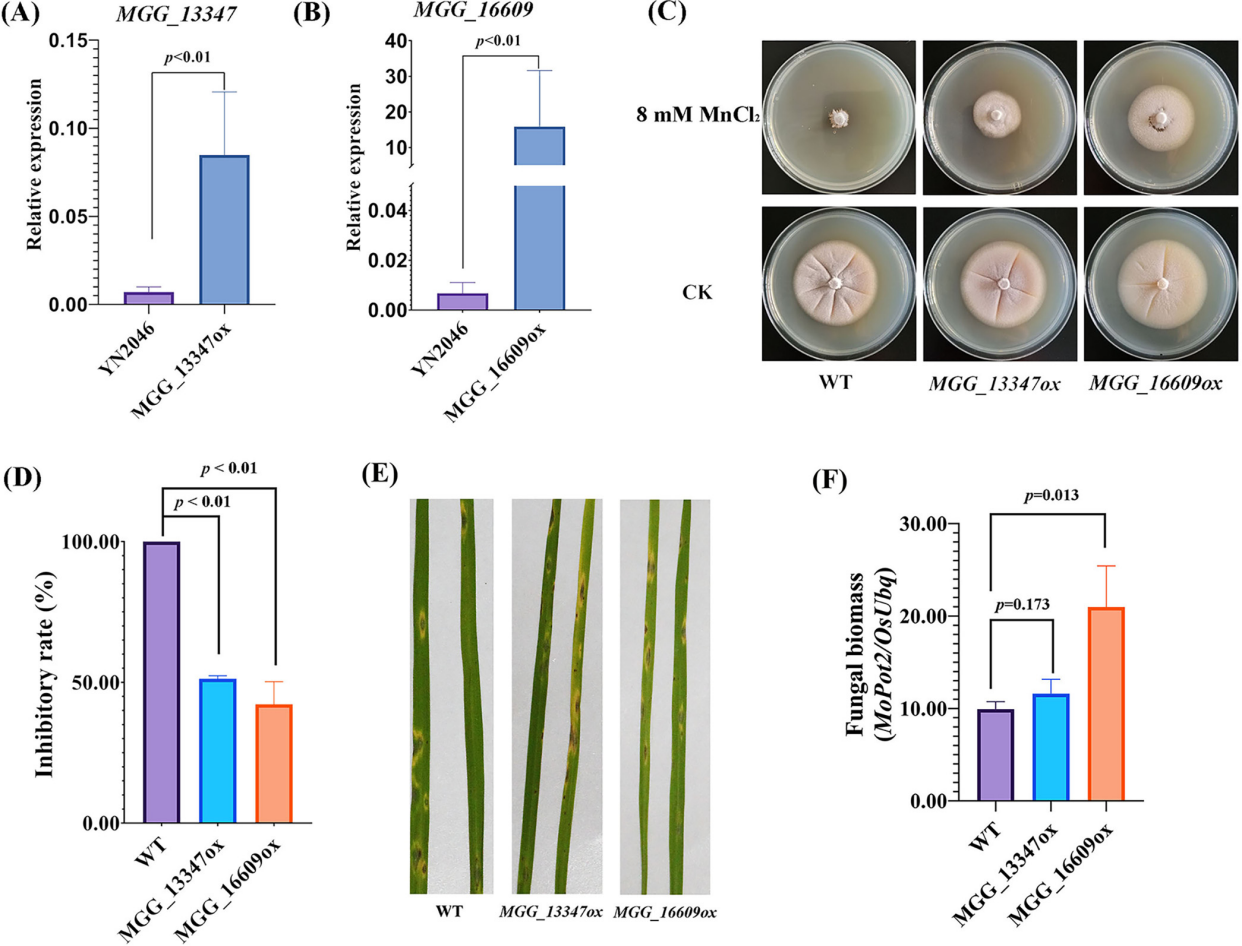
Overexpression of *MGG_13347* and *MGG_16609* enhanced tolerance to Mn^2+^ toxicity. The expression of *MGG_13347* (A) and *MGG_16609* (B) in WT (YN2046) and overexpression mutants. The phenotypes of *MGG_13347* and *MGG_16609* overexpression mutants in response to MnCl_2_ (C). The inhibitory rate of different strains with Mn^2+^ treatment (D). Lesions caused by different strains (E). The fungal biomass developed by different strains (F). These experiments were repeated three times. Statistically significant differences were calculated by Student’s *t* test. Error bars represent the means ±SE.

To further confirm the roles of mutant loci in the YN2046 with Mn sensitivity, we transformed the allele genes that were cloned from YN125 to YN2046 with the strong constitutive promoter *RP27*. However, only *MGG_13347* and *MGG_16609* were successfully overexpressed in YN2046 (Fig. 4A and B). Intriguingly, the overexpression of the two genes increased the tolerance to excessive Mn (Fig. 4C and D), indicating that the mutant loci among these two genes resulted in Mn sensitivity in YN2046, and this was probably due to downstream transcription not being activated. Moreover, we found that lesions with the 5th degree were formed on leaves that were inoculated with the three strains (Fig. 4E). The *MGG_16609* overexpression mutant yielded a significantly higher fungal biomass than that of the other two strains (Fig. 4F). According to previous transcriptomic results for the interaction between rice and blast fungus, we found that these two genes exhibit higher expressions during infection (29), implying that these two genes are activated in pathogenicity. Importantly, our results suggested that the overexpression of *MGG_16609* enhanced the pathogenicity in M. oryzae.

### Transcriptome analyses were performed to characterize the overexpression of *MGG_13347* and *MGG_16609* in regulating downstream genes.

**FIG 5:**
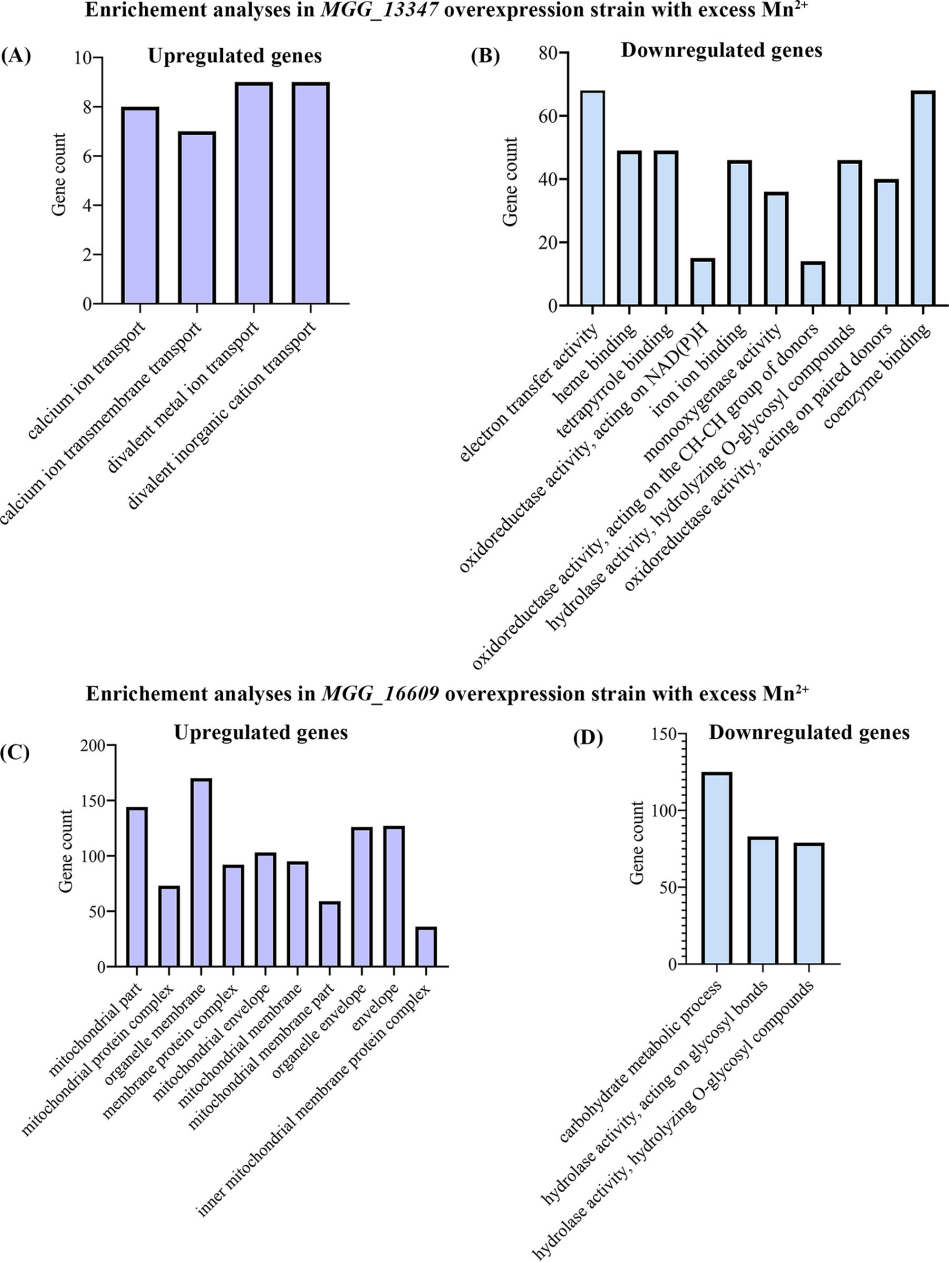
The GO enrichment of DEGs in the *MGG_13347* and *MGG_16609* overexpression strains under the Mn^2+^ treatment. The GO enrichment analyses for upregulated (A) and downregulated (B) genes in *MGG_13347* overexpression strains under the Mn^2+^ treatment. The GO enrichment analyses for upregulated (C) and downregulated (D) genes in *MGG_16609* overexpression strains under Mn^2+^ treatment. The top 10 GO terms with the highest *P value*s are presented in panels B and C.

In contrast to the previous results, no annotation was found for *MGG_13347* and *MGG_16609*. Through sequence alignment and secondary structural analyses, it was determined that *MGG_13347* might encode a membrane protein (Fig. S7A to C). We also predicted the crystal structures using AlphaFold2. The structure of MGG_16609 is similar, and there are moderate differences between the structures of the two SNP alleles (Fig. S7E and F). Due to the lack of annotation information for *MGG_13347* and *MGG_16609*, we performed RNA sequencing to analyze the regulating genes that are downstream in the *MGG_13347* and *MGG_16609* overexpression strains under Mn stress according to previous protocols. The transcriptomic results showed that more DEGs were regulated in the two overexpression strains under Mn treatment than in YN2046 (Fig. 5). For GO annotation, upregulated DEGs were significantly enriched in calcium ion transport-related terms (Fig. 5A), and downregulated DEGs were significantly enriched in oxidoreductase-related terms in the *MGG_13347* overexpression strain that received the Mn treatment (Fig. 5B). Moreover, upregulated DEGs were significantly enriched in mitochondrial-related oxidative phosphorylation-related terms (Fig. 5C), and downregulated DEGs belonged to hydrolase activity-related terms in the *MGG_16609* overexpression strain under Mn stress (Fig. 5D). We also found that the GO terms in the *MGG_13347* and *MGG_16609* overexpression strains under normal conditions were different from those under Mn treatment (Fig. S8). These results indicated that these DEGs in the overexpression strains were due to the Mn treatment rather than the overexpression of the candidate genes. Moreover, we provided compelling evidence for the expression of the interacting genes that are generated by WGCNA for *MGG_13347* and *MGG_16609* (Fig. S9). We found that 82 *MGG_16609-*interacting and 207 *MGG_13347*-interacting genes were not expressed in the YN2046 with Mn treatment and were reactivated in the overexpression strains (Table S7). These results indicated that SNPs in *MGG_13347* and *MGG_16609* were associated with the inability of nonresponsive genes to cope with Mn toxicity in YN2046.

### Overexpression of two genes alters enzyme activities and metabolite levels.

**FIG 6:**
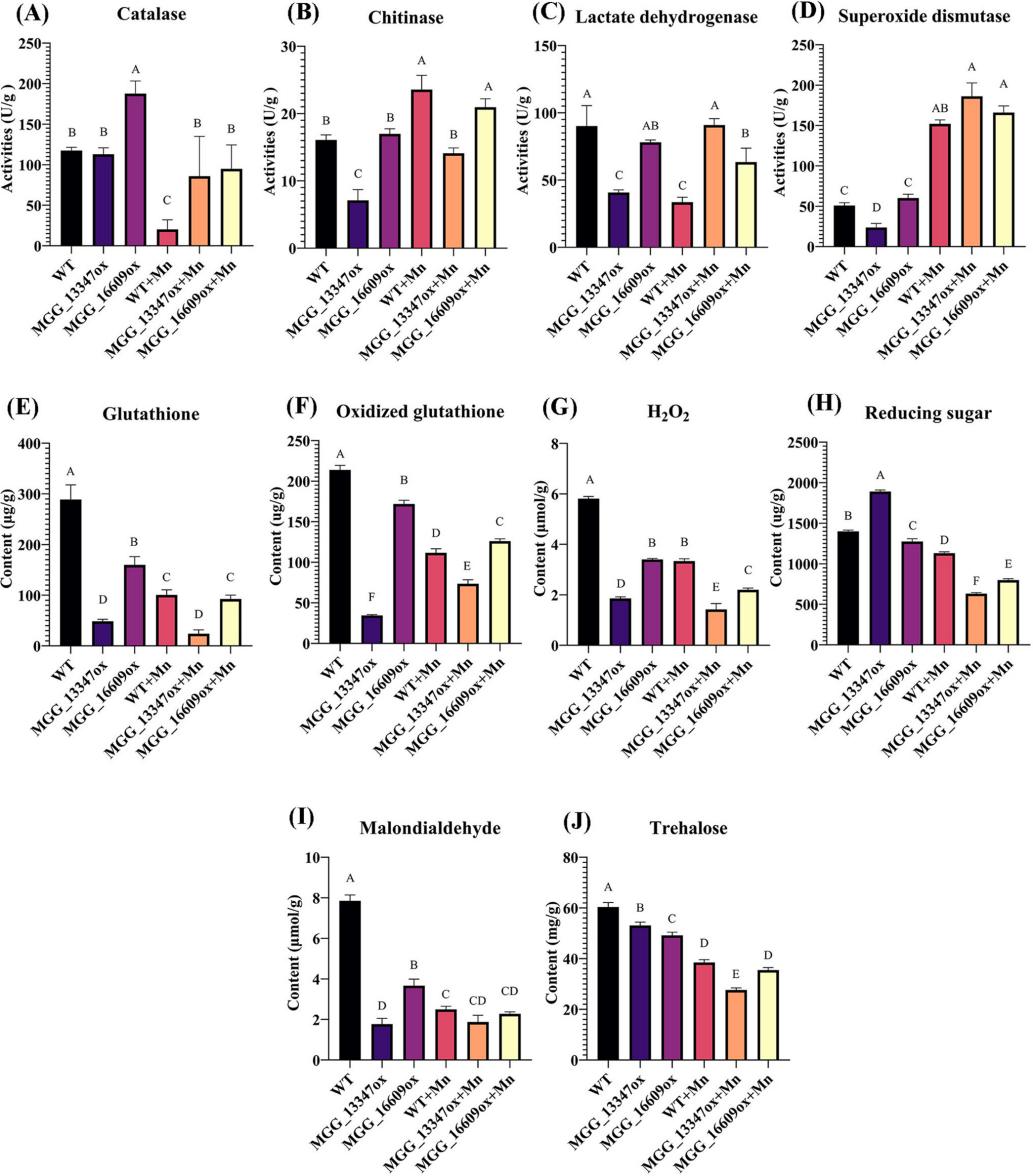
The variations in enzyme activities and metabolite contents in different strains treated with excessive Mn^2+^. The activities of catalase (A), chitinase (B), lactate dehydrogenase (C), and superoxide dismutase (D) and the contents of glutathione (E), oxidized glutathione (F), H_2_O_2_ (G), reducing sugar (H), malondialdehyde (I), and trehalose (J). These experiments were repeated three times. Statistically significant differences were calculated by ANOVA and Duncan’s test. Error bars represent the means ±SE. The different letters above each bar graph indicate significant differences (*P < *0.01) among various treatments.

It is well recognized that excessive Mn can affect the activities of antioxidation enzymes in M. oryzae. Accordingly, we assessed enzyme activities and the contents of metabolites in the present study (Fig. 6). We found that among the YN2046 (WT), *MGG_13347*, and *MGG_16609* overexpression mutants under Mn treatment, the activities of chitinase and SOD were increased, and the contents of H_2_O_2_, reducing sugars, and trehalose were reduced. The catalase and lactate dehydrogenase activities in the *MGG_13347* and *MGG_16609* overexpression mutants were higher than those in the WT with Mn treatment. Although the overexpressed mutants exhibited tolerance to Mn toxicity, the glutathione, oxidized glutathione, and trehalose contents were lower in the *MGG_13347*-overexpressing mutant than in the WT and the *MGG_16609* overexpression mutant. After Mn treatment, the malondialdehyde contents were not altered in the MGG_13347-overexpressing mutant. Moreover, we found that these enzymes exhibited different transcription modes in different strains under Mn stress (Table S8), suggesting that the regulation of *MGG_13347* might be different from that of *MGG_16609* in response to Mn toxicity at the enzyme activity and metabolite levels.

## DISCUSSION

Mn is an essential trace metal that is a necessary cofactor for diverse enzymes, such as MnSOD and laccase. However, any deficiency or excess of Mn can reportedly facilitate cellular dysfunction (18, 30). Thus, it is essential to maintain Mn homeostasis through Mn-associated transporters and metabolites, which have been identified in plants, animals, and microbes (10, 24, 25, 31). Moreover, many transcript regulation-related genes, such as Mn-binding transcription factors and an Mn-binding riboswitch, have been documented to induce the expression of downstream Mn transporters in some bacteria (32). Many transcription factors in M. oryzae have been reported to be regulated by Mn^2+^ toxicity, and some of these transition factors are also induced during fungal development and infection (33), implying that Mn^2+^ could activate the reprogrammable transcriptome that is involved in intricate cellular functions. In a previous study, we identified a cytochrome P450 gene, *MoMCP1*, and deduced the gene expression network through which *MoMCP1* regulates Mn^2+^ toxicity in M. oryzae (20). Herein, we tried to elucidate other transcriptomic features in the Mn^2+^-sensitive strain YN2046 to explore the mechanism underlying the response of M. oryzae to excess Mn^2+^.

### The different number of DEGs might indicate heavy metal tolerance.

To better analyze the gene expression network in the Mn^2+^-sensitive strain YN2046, we reviewed the literature on the published transcriptome data of M. oryzae under the same conditions (Fig. 2A and B). It was reported that the expression of trace element homeostasis, metabolic pathways, organelles, and signal transition-related genes are responsible for Mn^2+^ stress in M. oryzae (20). However, many genes were not regulated in YN2046 (Fig. S1), indicating that the absence or mutation of Mn^2+^-sensing transcription factors or essential genes leads to the inactivation of downstream gene expression. Moreover, organisms that are tolerant to heavy metals often exhibit stronger transcriptomic regulation. In contrast, a study reported 2286 DEGs in a cadmium-tolerant cultivar and 111 DEGs in a sensitive cultivar, and more genes were involved in antioxidant defense, phytohormones, and the cell cycle in the tolerant cultivar (34). Moreover, a high accumulation of Cd in sweet sorghum induced a more significant number of DEGs that were enriched in GO terms and KEGG pathways (35). Recent evidence suggests that Pleurotus fungi have great potential for heavy metal mycoremediation, and different fungi with different heavy metal bioremediation abilities possess DEGs with significantly different expression levels in response to cadmium exposure (36). Thus, quantitative differences in transcriptomic reprogramming genes indicate that YN2046 and YN125 have different abilities to cope with Mn toxicity.

### Strain unique specific modules associated with different phenotypes with Mn treatment.

To further explore the specific expression pattern in YN2046 that is associated with Mn^2+^ toxicity, WGCNA was performed to identify the modules affected by Mn^2+^. Over the years, WGCNA has been extensively applied in studies on the responses of plants to heavy metals (37, 38), the fungal development of plants and the ability of plants to utilize nutrition (38–40). In the present study, WGCNA was used to construct interaction networks in YN125 and YN2046. The high modularity observed suggested that the nodes within a module were tightly correlated. A small number of nodes and edges were found in YN2046 with Mn^2+^ treatment, while the average degree and modularity were more significant in YN125 than in YN2046 (Fig. 3). Moreover, we found many hypothetical protein-coding genes, and WGCNA could be used to speculate the functions of these proteins.

For the analysis of significant trait-related modules in YN2046, the turquoise module contained the most nodes, but no significant relationship was found between the trait and the turquoise module. Most DEGs that were associated with the black module in YN125 and YN2046 were downregulated (Fig. S6C). Enrichment analyses indicated that this module was mainly involved in nutrition utilization and integral component of membrane. Allantoate permease, dihydrodipicolinate synthase, general amino acid permease AGP2, thymine dioxygenase, aminopeptidase Y, amidase, lipase 2, high-affinity glucose transporter, tissue alpha-L-fucosidase, 2-amino-3-carboxymuconate-6-semialdehyde decarboxylase, alpha-galactosidase, and unsaturated glucuronyl hydrolase were downregulated in the two isolates, and these genes were involved in carbohydrate and nitrogen metabolism, indicating that Mn toxicity disrupts the intake of carbon and nitrogen in rice blast fungus. Similar to the turquoise module, the magenta module that contained genes showed similar expression patterns in YN2046 and YN125. However, only five genes were expressed in YN2046, including neutral ceramidase, cytochrome P450 monooxygenase, and three hypothetical proteins. Ceramidases play essential roles in ceramide metabolism and mediate polarized growth in Aspergillus nidulans (41) and have been documented in zebrafish with Cd^2+^ treatment (42). Many cytochrome P450 genes have been associated with heavy metal resistance (43) and have been used as biomarkers to characterize specific heavy metals (44). Importantly, specific downregulation of cytochrome P450 monooxygenase may cause Mn sensitivity in YN2046. For the yellow module in YN2046, 69% of DEGs (29) were annotated as hypothetical proteins, 38% of which (16) were only expressed with Mn treatment. Furthermore, the DEGs encoding secreted glucosidase, short-chain dehydrogenase/reductase, PHO85 cyclin-1, and ThiJ/PfpI family protein were expressed in YN2046. An increasing body of evidence suggests that in heavy metal-contaminated soil, the activities of β-glucosidase can reflect soil health (45, 46). For the green module, we found that pectate lyase and chitin synthase 2-related genes were downregulated. Pectate lyases are widely acknowledged to be used for plant cell wall degradation in phytopathogens (47), while pectic materials are promising adsorbents for heavy metals (48). In this regard, fungal chitin has been identified to be involved in heavy metal tolerance. Recent research has substantiated the potential of the fungal chitin-glucan nanopapers that are derived from *Agaricus bisporus* on heavy metal filters (49). This finding suggests that lower expression of chitin synthase 2 might cause lower chitin content and increase the susceptibility of YN2046 to Mn toxicity.

Enrichment analysis of the purple module in YN2046 showed that cell cycle-related genes were regulated in YN2046 with Mn treatment, and this result was consistent with the KEGG results for all DEGs of YN2046 (Fig. S3). Thus, DEGs in the purple module may play a critical role in Mn sensitivity in YN2046. It is well established that the cell cycle controls the duplication of DNA and the segregation of newly duplicated genomes into two daughter cells to regulate cell proliferation. Importantly, Mn^2+^ toxicity that disrupts the expression of cell cycle genes has been reported in other organisms (17). Among cell cycle DEGs, two DNA replication licensing factors, Mcm3 and Mcm7, are reportedly involved in the formation of minichromosome maintenance complexes and DNA replication licensing from late mitosis until the end of the G1 phase. DNA replication licensing ensures that genome duplication occurs properly during each cell cycle, and when the expression of replication licensing genes is aberrant, it can induce replication stress (50). Moreover, it has been reported that chromosome condensation genes engage and link the chromatin fibers of eukaryotic genomes (51, 52). In the present study, two chromosome condensation genes were also downregulated, implying that the structural transition of chromosomes might be disrupted by excess Mn^2+^. Another cell cycle-enriched gene that encodes a TTK protein kinase was also suppressed in YN2046 under excess Mn^2+^ treatment. TTK is a critical regulator in anaphase and controls the spindle assembly checkpoint to maintain genomic integrity. A downregulated gene that encodes separin has been reported to act as a protease to cleave a cohesion subunit and segregate chromosomes in anaphase (53). However, some cell cycle-related genes play dominant roles in tumorigenesis (54), and Mn complexes can exhibit anticancer activities (55, 56). A recent result substantiated that Mn is essential in antitumor immune responses via the cGAS-STING pathway (57). Therefore, a better understanding of the relationships among Mn, tumorigenesis, and the cell cycle might provide novel strategies for tumor therapy. However, it is difficult to distinguish whether excess Mn^2+^ attacks chromosomes directly or induces cell stress, such as oxidative stress, to disrupt DNA replication; therefore, further research using biochemistry and fluorescence imaging technologies is warranted.

### Alleles with SNPs inhibit the expression of downstream genes.

Compared with YN125, YN2046 contained fewer DEGs with Mn treatment. We hypothesize that this phenomenon might be attributed to a lack of excessive Mn sensing regulators or uncorrected translation proteins. In this study, during WGCNA, the turquoise and brown modules were significantly related to the YN125 with Mn treatment. Thus, we aligned the highly expressed gene sequences in YN125. Among the six genes, four genes were identified with SNPs in their upstream and ORF regions (Table 1). We cloned these four genes from YN125 with a constitutive *RP27* promoter and transformed them into YN2046. *MGG_13347-* and *MGG_16609-*overexpressing mutants were obtained, enhancing the tolerance to Mn toxicity in YN2046 (Fig. 4). These results demonstrated that the SNPs in the promoter and ORF regions might ensure that gene expression is incorrect and might induce Mn sensitivity in YN2046.

As determined by the transcriptomic analyses of *MGG_13347-* and *MGG_16609-*overexpressing mutants under Mn toxicity, Ca^2+^ transport-related genes were significantly enriched (Fig. 5A). There is overwhelming evidence that supports the relationship between Mn toxicity and Ca^2+^ activity. For instance, Mn-induced neurotoxicity is involved in calcium signaling and mitochondrial calcium uniporter (58, 59). In wheat, higher Mn concentrations inhibit the uptake of phosphorus and magnesium but increase the Ca proportion in the root apoplast (60). These results indicate that calcium plays a detoxification role in response to excess Mn. Based on interacting networks, we found that all genes encoding ribosomal structure proteins were downregulated and mediated by *MGG_13347* (Table S8). In contrast, the expression levels of most ribosome-related genes were upregulated in maize roots in response to lead stress (61). In Saccharomyces cerevisiae, the zinc transcriptional regulatory element regulates zinc homeostasis and ribosome biogenesis (62). Excess heavy metals can bind proteins to disrupt the correct conformation and induce the accumulation of toxic proteins. Our results found that the downregulation of all ribosome morphogenesis-related genes that was mediated by *MGG_13347* could reduce protein synthesis to prevent the accumulation of these abnormal proteins, which is conducive to cellular dysfunction.

The transcriptome enrichment results between *MGG_16609* and *MGG_13347* were different, suggesting the presence of unique expression patterns between the *MGG_13347* and *MGG_16609* overexpression strains. The upregulated mitochondria-related genes in the *MGG_16609* overexpression strain were enriched in mitochondrial structure, ATP activity, and energy production following Mn treatment (Fig. 5C). An increasing body of evidence suggests that Mn decreases energy metabolism *in vivo* and *in vitro*, including decreased mitochondrial enzyme activity, membrane potential, and ATP production. Kiran Kalia et al. reported that mitochondria weakly bind with Mn (63). Furthermore, DMT1, a divalent metal transporter that is responsible for Mn uptake, has been documented in mitochondria (64). We identified a cytochrome P450 gene, *MoMCP1*, in M. oryzae and found that a large number of mitochondria and oxidative phosphorylation-related genes were downregulated in △*Momcp1* under Mn toxicity (20). For *MGG_16609-*regulating genes (Table S8), many DEGs involved in oxidoreduction were highly upregulated, including cytochrome P450 52A12 (FC = 3.03), P450 monooxygenase (FC = 0.638), FAD monooxygenase (FC = 4.96), and NADPH-dependent 1-acyldihydroxyacetone phosphate reductase (FC = 5.91). Oxidoreductase-related GO terms could be found in many transcriptomic results and were associated with heavy metal tolerance in plants, animals, and fungi (65–68). It is widely thought that oxidoreductase activity can be used to indicate the degree of heavy metal pollution in different environments (69). In the present study, we found that oxidoreductases with high expressions that are regulated by *MGG_16609* play specific roles in M. oryzae under Mn stress. The RNA sequencing results further confirmed the functions of *MGG_13347* and *MGG_16609* in alleviating Mn toxicity through the regulation of calcium and mitochondria-related pathways.

### Overexpression mutants regulate tolerance through changes in enzyme activities and metabolite levels.

Heavy metal stress often causes enzyme dysfunction and metabolic disturbance. We assessed the activities of four enzymes and the contents of six metabolites to explore the mechanism of Mn tolerance in the two overexpression strains (Fig. 6). Catalase and superoxide dismutase are regarded as antioxidant enzymes. Catalase mainly catalyzes hydrogen peroxide to produce H_2_O and O_2_, while superoxide dismutase is responsible for alleviating superoxide stress. With Mn treatment, the changes in CAT and SOD activity in the three strains were consistent with that in the literature (20). However, we found that chitinase activity was increased in all three strains, implying that chitinase might play an essential role in Mn detoxification. Chitinases are widely acknowledged to be responsible for the remodeling of chitin, which is a major fungal cell wall component. In rice blast fungus, deleting a chitinase gene, *MoChia1*, caused an abnormal deposition of chitin, decreased appressorium formation, and attenuated virulence (70). Overwhelming evidence suggests that different kinds of heavy metal stress can induce the accumulation of various chitinase isoforms in plants (71), indicating that there is a direct association between chitin isoforms and heavy metals. Moreover, chitin-related compounds can bind heavy metals (49), and increased chitinase activities can favor the scavenging of metals that bind cell walls. We found that lactate dehydrogenase activities were decreased in YN2046 with Mn treatment and increased in the *MGG_13347*-overexpressing strain. Lactate dehydrogenase catalyzes the oxidation of L-lactate to pyruvate and is considered a biosensor for heavy metals (72). In teleost fish, many heavy metals, such as cadmium, mercury, lead, and arsenic, cause severely decreased lactate dehydrogenase activity (73). In contrast, we found that lactate dehydrogenase activities increased in the *MGG_13347*-overexpressing strain and were not significantly changed in the *MGG_16609*-overexpressing strain, implying that excessive Mn disrupted lactate dehydrogenase activities in YN2046. Ample evidence substantiates that trehalose plays protective roles in plants and animals in response to heavy metals and exhibits antioxidant, antiapoptotic, and autophagy properties (74–76). In M. oryzae, trehalose-6-phosphate synthase gene mutants exhibited a much lower content of trehalose and failed to generate pressure in appressoria (77). However, in the present study, Mn toxicity resulted in lower trehalase contents in all three strains, implying that the overexpression of two genes does not alter trehalase levels as a tolerance mechanism against excessive Mn.

However, we found that the overexpression of *MGG_13347* and *MGG_16609* conferred tolerance to Mn^2+^ toxicity in M. oryzae through comparative transcriptome and SNP analyses. Interestingly, the SNP in the promoter region of *MGG_13347* could not be recognized by transcription factors, while the SNPs in *MGG_16609* coding regions might result in an incorrect expression, and downstream gene expression could not be activated. Thus, a weaker transcript response was observed in YN2046, and fewer genes were regulated in response to Mn toxicity. However, there was no valuable information for the annotation of these two genes through sequence alignment and second structural analyses (Fig. S7), implying the need for further studies to uncover the functions of these genes. Indeed, omics approaches that are combined with genetics can be used to explore the functions of these genes. With the increase in published studies on the genomic sequences of M. oryzae in recent years, rice blast fungi have been used for Mn toxicity research similar to yeast (78). Indeed, the comprehensive genetic resources of M. oryzae combined with multiomics analyses, such as genome-wide association studies (GWAS) and proteomics, can be used to study preferred traits and disease models.

## MATERIALS AND METHODS

### Strain materials and growth conditions.

The M. oryzae strains were collected from rice lesions in the Yunnan area through single spore isolations. Solid complete medium (CM, 0.6% yeast extract, 0.6% casein hydrolysate, 1% sucrose, and 1.5% agar) was used for fungal growth under excess Mn^2+^ (8 mM). Liquid CM was used to measure the dry weight of the mycelial, and detailed protocols were based on previous reports (20). For inoculation, 21-day growing rice seedlings were inoculated by spraying with the spore. The spore suspension of each strain was adjusted to 1 × 10^5^ spores/mL. The inoculated materials were maintained in a dark environment with a high humidity at 28°C. One day after the inoculation, the materials were transferred to a growth chamber. The disease index was calculated at 7-days postinoculation, and fungal growth was estimated according to Kawano’s methods (79).

### Construction of the overexpression strain.

The vector pDL2 with the strong constitutive *RP27* promoter was used to construct the overexpression vector. The full-length gene coding region was amplified from YN125 genomic DNA and was inserted into pDL2 to generate recombinant vectors using the yeast gap repair approach. The correct recombination vector was confirmed by Sanger sequencing and was then transformed into M. oryzae YN2046 protoplasts with PEG-mediated transformation. The positive transformants were selected and confirmed by PCR and Sanger sequencing.

### RNA sequencing and WGCNA.

The mycelia were harvested from liquid CM with 2 mM Mn^2+^ for 2 days, and the untreated mycelia were used as a negative control. Each treatment consisted of three biological samples. The mycelia were dried quickly by filter paper and frozen in liquid nitrogen. Total RNA was extracted by an RNeasy Plus minikit (Qiagen, Hilden, MA, USA) from each sample and was prepared for RNA sequencing and qRT–PCR.

The libraries were subjected to sequencing on the Illumina HiSeqTM 4000 platform by Novogene Co. (Beijing, China). The genome of Magnaporthe oryzae 70-15 was used as the reference genome and was downloaded at NCBI (https://www.ncbi.nlm.nih.gov/genome/62?genome_assembly_id=22733, accessed on 31 March 2016). The R package DESeq2 was used to identify DEGs between two different samples with a false discovery rate (FDR) ≤0.05. GO functional enrichment and KEGG pathway analyses were carried out by Goatools (https://github.com/tanghaibao/Goatools) and KOBAS (http://kobas.cbi.pku.edu.cn/home.do).

The R package WGCNA was used to build the gene coexpression network. A total of 18 transcriptome samples (six new samples were obtained from this study and 12 samples were from published studies) were analyzed. The gene expression matrix was constructed using log_2_(FPKM + 1), and the transcripts with a median absolute deviation (MAD) >0.1 were selected. The soft threshold power was 20 with R^2^>0.8 and mean connectivity <100 according to scale-free topology criteria. The cluster dendrogram was based on topological overlap values for all transcripts, and the modules with highly coexpressed transcripts were merged using a cutoff value of 0.25. The correlation between different modules was presented by a TOM plot. The visualized network plots of different modules were generated by Gephi software.

The Pearson correlation coefficients were calculated to assess the relationship between module eigengenes and traits. Trait-associated modules with *P* < 0.05 were selected, the correlations between module membership and gene significance were calculated, and genes with high values of module membership and gene significance were regarded as hub genes that regulate the trait in a module.

### Assays for enzyme activity and metabolite content.

The assay kits for catalase, chitinase, lactate dehydrogenase, superoxide dismutase, glutathione, oxidized glutathione, H_2_O_2_, reducing sugar, malondialdehyde, and trehalose were purchased from Beijing Solarbio Science & Technology Co., Ltd. The assay methods were conducted according to the instructions of the assay kits.

### SNP analyses.

The primers were designed by Primer 5.0 software according to the reference genome. The primer sequences are listed in Table S9. A 2 × Phanta Max Master Mix kit was used for target fragment cloning and sequencing by Sanger sequencing. MEGA 5.0 was used for sequence alignment.

### Protein sequence and structure analyses.

The protein sequence analyses were conducted by PSIPRED v4.0 (http://bioinf.cs.ucl.ac.uk/psipred/). The three-dimensional structures were predicted by AlphaFold2 supported by the BKUNYUN supercomputing platform. Pictures of the structures were generated by PyMOL.

### Data availability.

All raw sequencing data have been deposited into the NCBI Sequence Read Archive (SRA) database under the accession numbers PRJNA523930 and PRJNA642965.
