# Supplementary material for: Transcriptome Analysis and SNP Identification Reveal That Heterologous Overexpression of Two Uncharacterized Genes Enhances the Tolerance of Magnaporthe oryzae to Manganese Toxicity
Source: Microbiol Spectr. 2022 May 31;10(3):e02605-21. doi: 10.1128/spectrum.02605-21 (PMC9241697; doi:10.1128/spectrum.02605-21)
Supplement: SUPPLEMENTAL FILE 1 — Supplemental material. Download spectrum.02605-21-s001.pdf, PDF file, 1.5 MB [file spectrum.02605-21-s001.pdf]

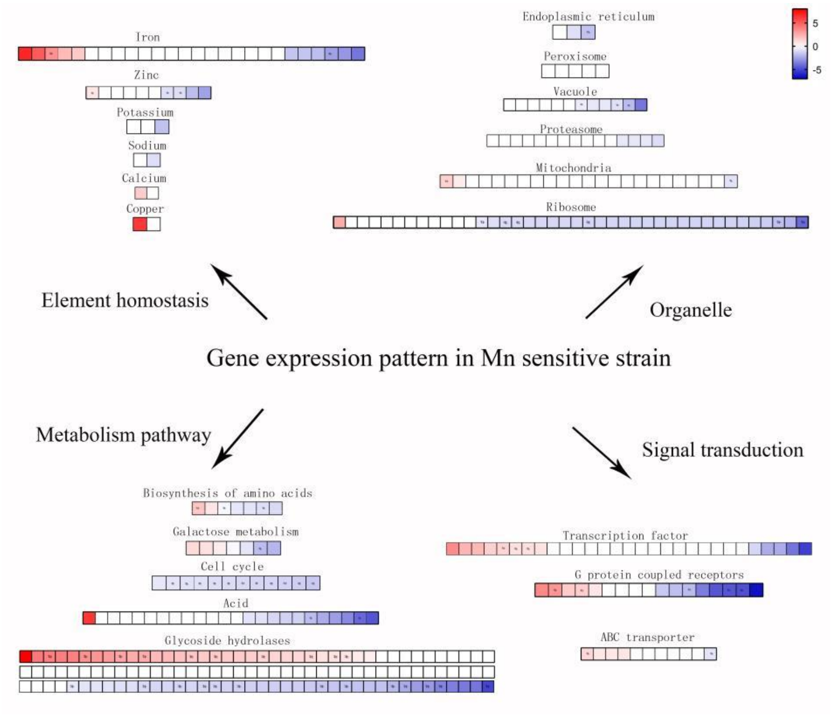

Figure S1. The characters of gene expression pattern in YN2046 with comparative transcriptomic analyses.

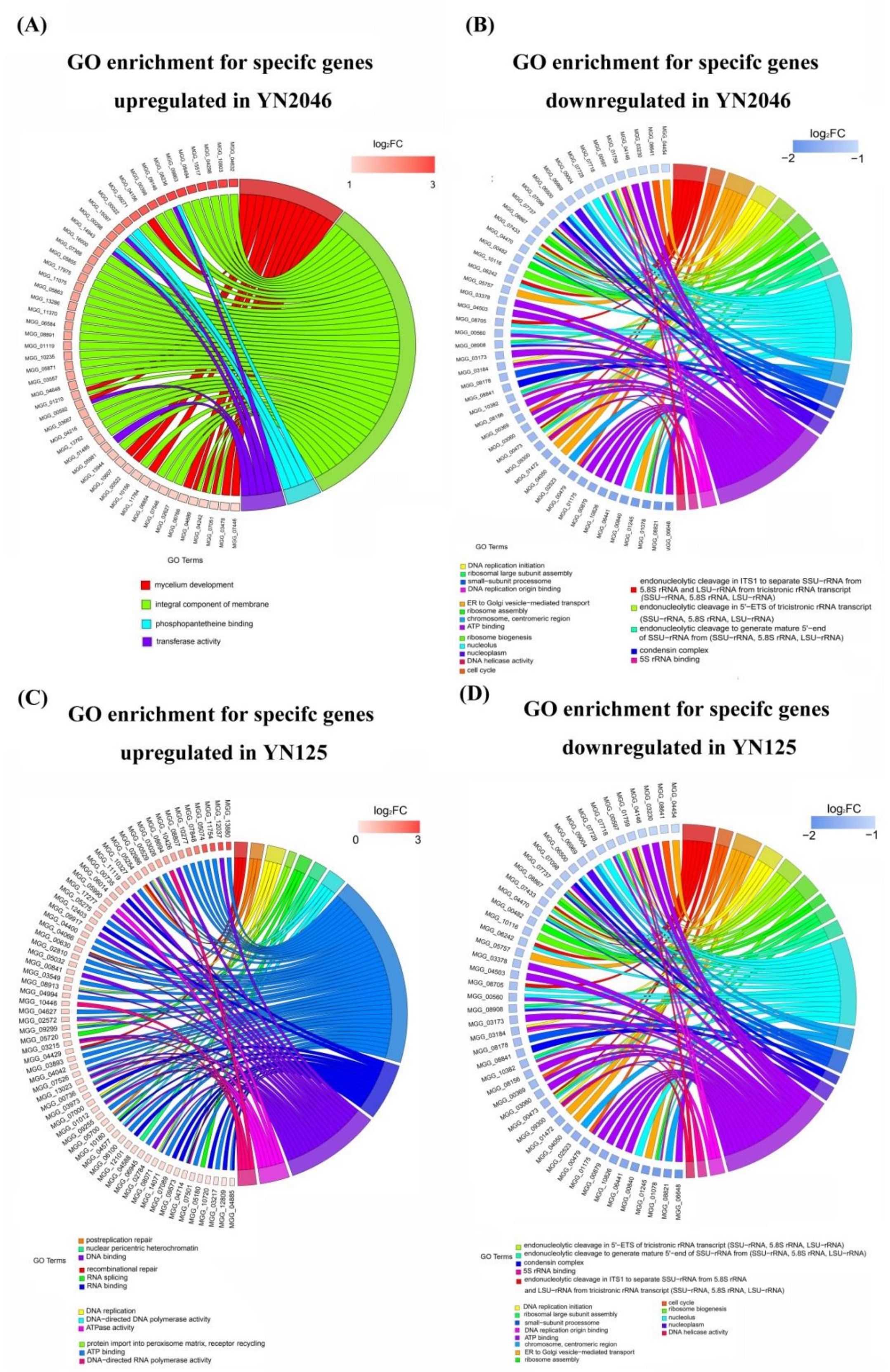

Figure S2. GO analyses of specific DEGs under excessive Mn^{2+} treatment in YN2046 and YN125, respectively. GO enrichment analyses of DEGs upregulated (A) and downregulated (B) specifically in

YN2046. GO enrichment analyses of DEGs upregulated (C) and downregulated (D) specifically in YN125.

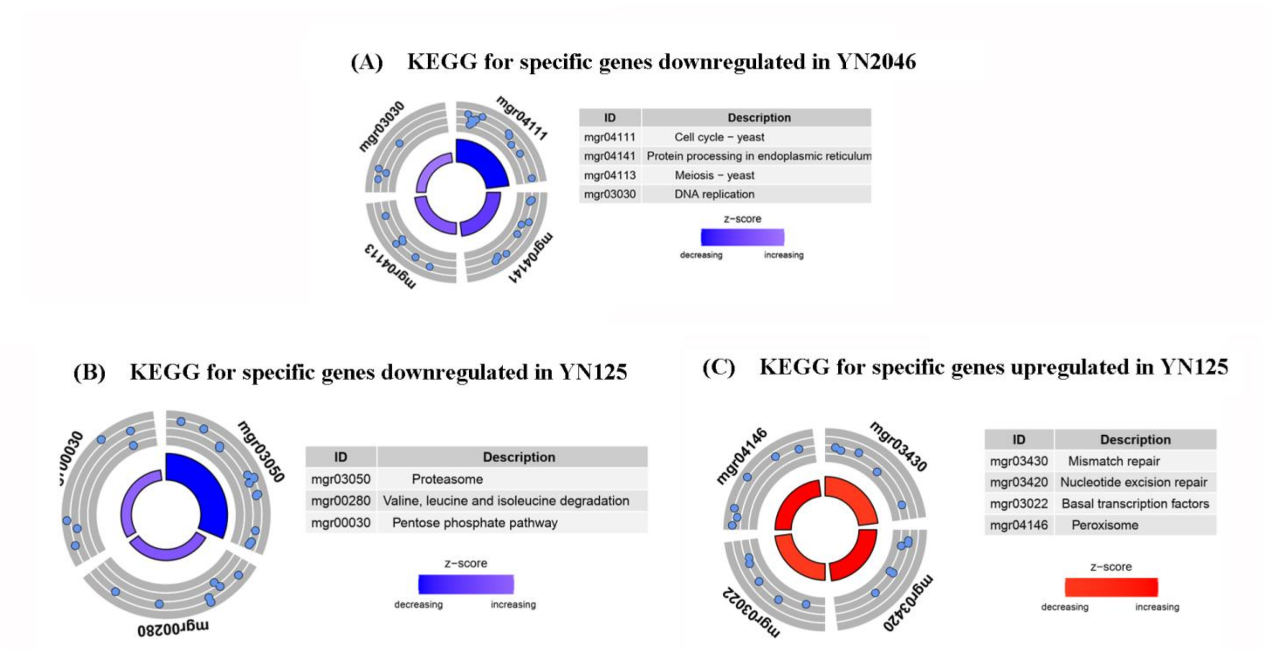

Figure S3. KEGG pathways for specific DEGs under excessive Mn^{2+} treatment in YN2046 and YN125, respectively. KEGG analyses of DEGs downregulated (A) specifically in YN2046. KEGG analyses of DEGs upregulated (B) and downregulated (C) specifically in YN125

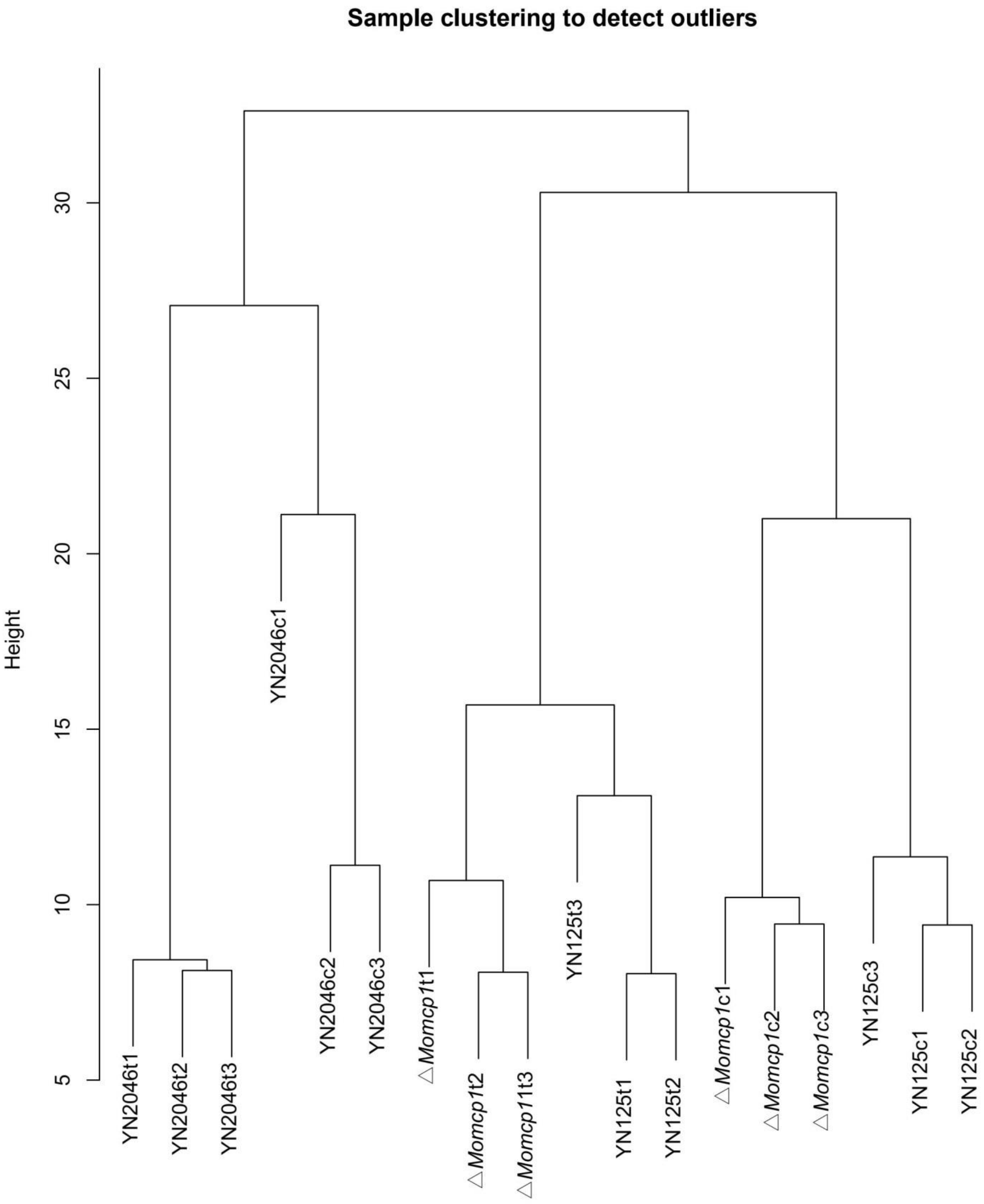

Figure S4. The cluster of different transcript samples used for WGCNA.

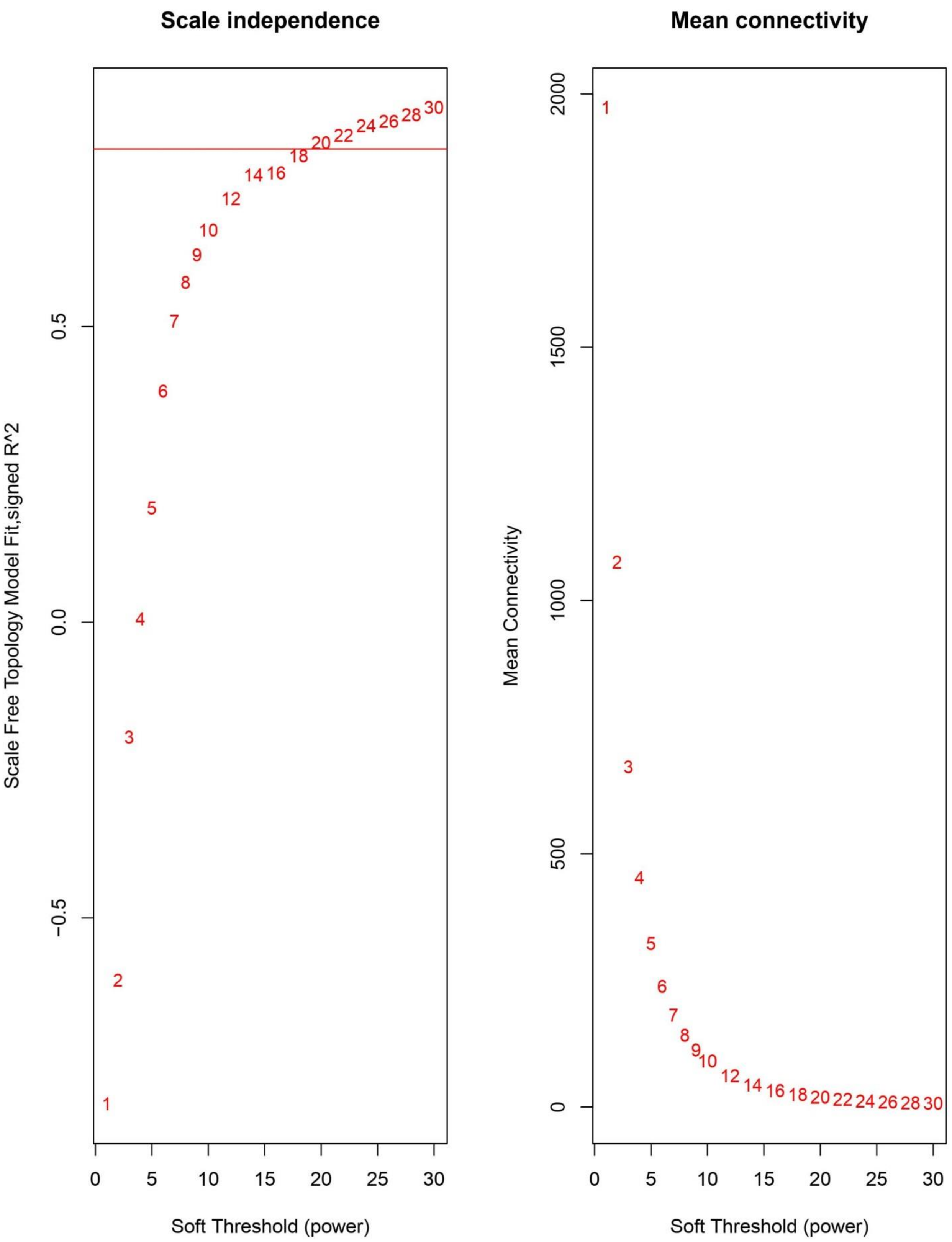

Figure S5. The scale independence and mean connectivity plots used for power screen.

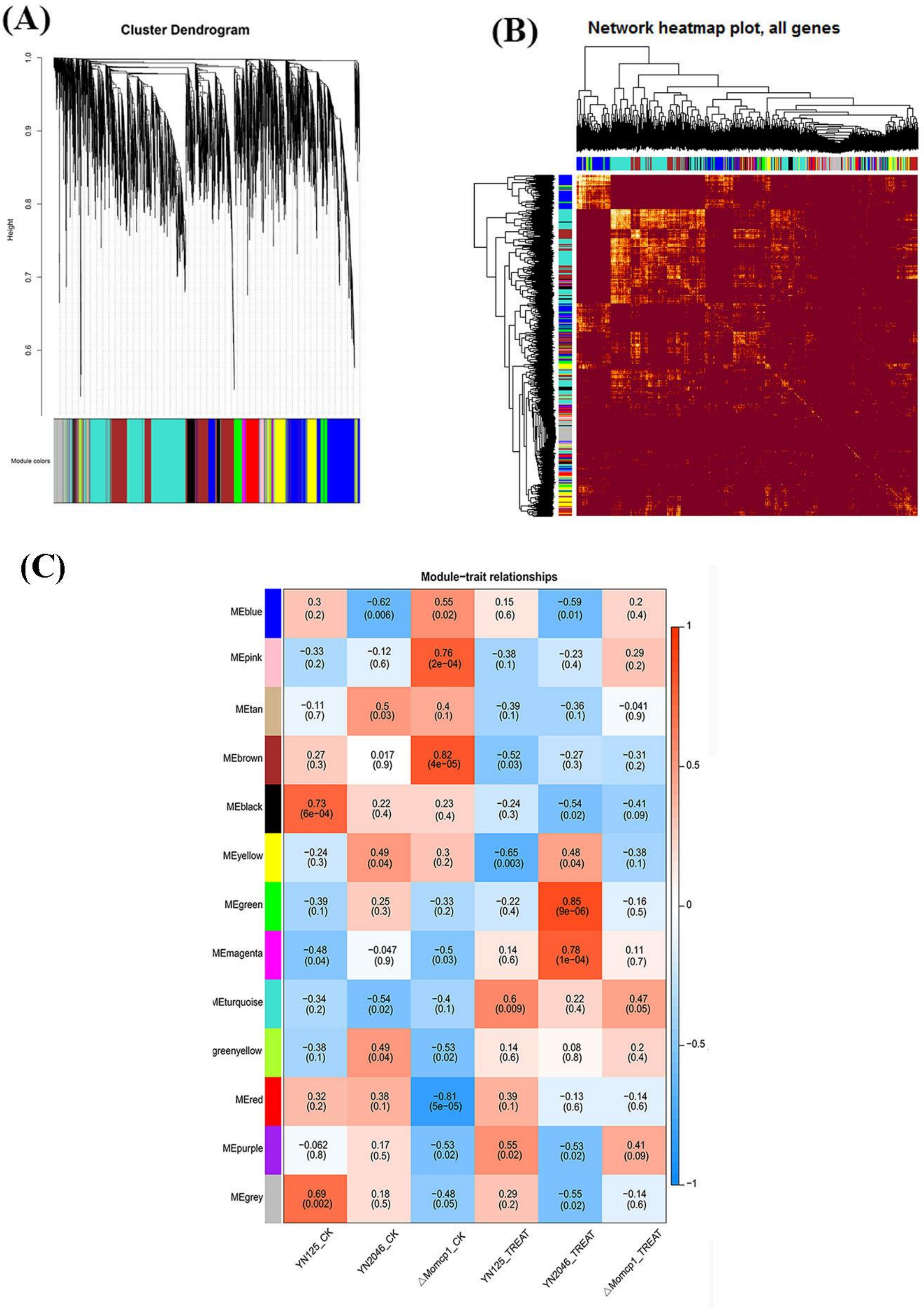
(A)
Cluster Dendrogram
Height
1.0
0.9
0.8
0.7
0.6
Module colors
(B)
Network heatmap plot, all genes
(C)
Module−trait relationships
MEblue
0.3 (0.2)
−0.62 (0.006)
0.55 (0.02)
0.15 (0.6)
−0.59 (0.01)
0.2 (0.4)
MEpink
−0.33 (0.2)
−0.12 (0.6)
0.76 (2e−04)
−0.38 (0.1)
−0.23 (0.4)
0.29 (0.2)
MEtan
−0.11 (0.7)
0.5 (0.03)
0.4 (0.1)
−0.39 (0.1)
−0.36 (0.1)
−0.041 (0.9)
MEbrown
0.27 (0.3)
0.017 (0.9)
0.82 (4e−05)
−0.52 (0.03)
−0.27 (0.3)
−0.31 (0.2)
MEblack
0.73 (6e−04)
0.22 (0.4)
0.23 (0.4)
−0.24 (0.3)
−0.54 (0.02)
−0.41 (0.09)
MEyellow
−0.24 (0.3)
0.49 (0.04)
0.3 (0.2)
−0.65 (0.003)
0.48 (0.04)
−0.38 (0.1)
MEgreen
−0.39 (0.1)
0.25 (0.3)
−0.33 (0.2)
−0.22 (0.4)
0.85 (9e−06)
−0.16 (0.5)
MEmagenta
−0.48 (0.04)
−0.047 (0.9)
−0.5 (0.03)
0.14 (0.6)
0.78 (1e−04)
0.11 (0.7)
MEturquoise
−0.34 (0.2)
−0.54 (0.02)
−0.4 (0.1)
0.6 (0.009)
0.22 (0.4)
0.47 (0.05)
greenyellow
−0.38 (0.1)
0.49 (0.04)
−0.53 (0.02)
0.14 (0.6)
0.08 (0.8)
0.2 (0.4)
MEred
0.32 (0.2)
0.38 (0.1)
−0.81 (5e−05)
0.39 (0.1)
−0.13 (0.6)
−0.14 (0.6)
MEpurple
−0.062 (0.8)
0.17 (0.5)
−0.53 (0.02)
0.55 (0.02)
−0.53 (0.02)
0.41 (0.09)
MEgrey
0.69 (0.002)
0.18 (0.5)
−0.48 (0.05)
0.29 (0.2)
−0.55 (0.02)
−0.14 (0.6)
YN125_CK
YN2046_CK
△Momcp1_CK
YN125_TREAT
YN2046_TREAT
△Momcp1_TREAT
1
0.5
0
−0.5
−1

Figure S6. WGCNA among different transcriptome samples. (A) Gene dendrogram of 18 samples and 13 modules with different colors obtained by WGCNA. (B) TOM plot for gene interacting network analysis. (C) The module and trait relationship among different transcript samples.

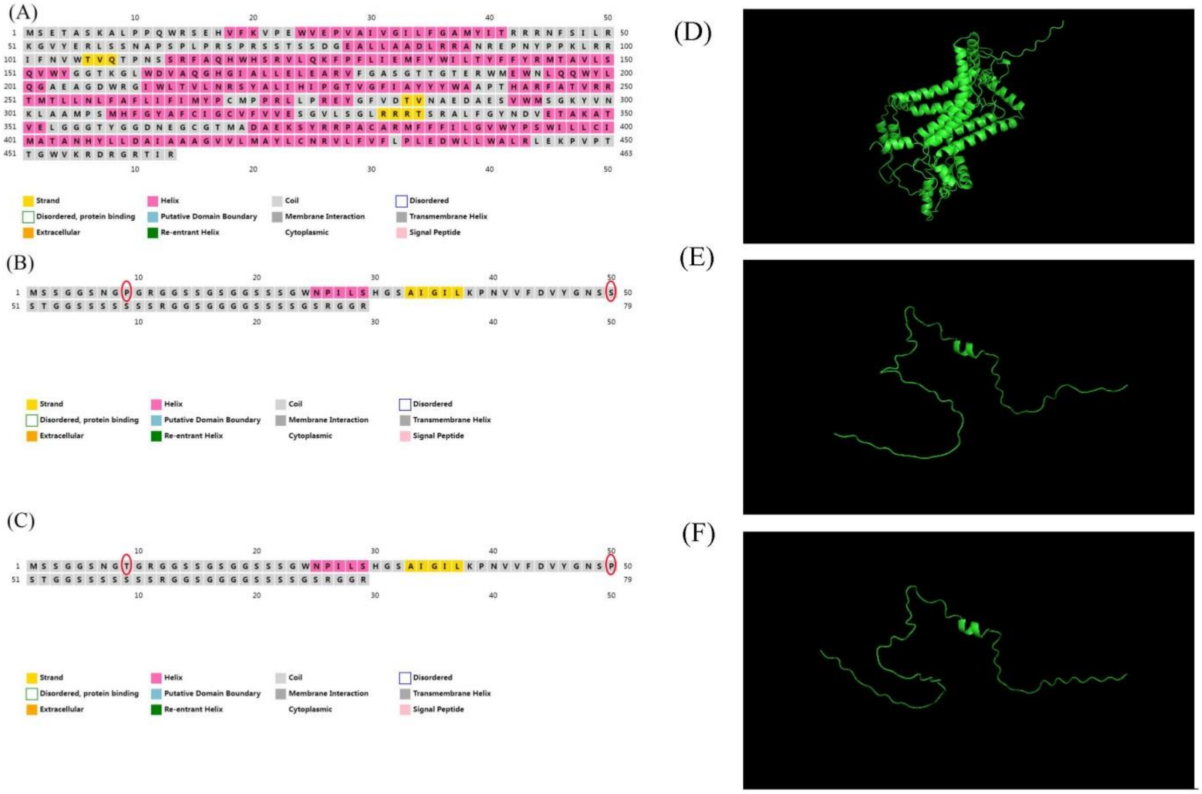

Figure S7. The second structure and three-dimension of MGG_13347 and MGG_16609. (A) The protein sequence of *MGG_13347* both in YN125 and YN2046. The protein sequence of MGG_16609 both in YN125 (B) and YN2046 (C). The three-dimensional structure predicted by Alphafold 2.0 of MGG_13347 (D), MGG_16609 in YN125 (E) and MGG_16609 in YN2046 (F).

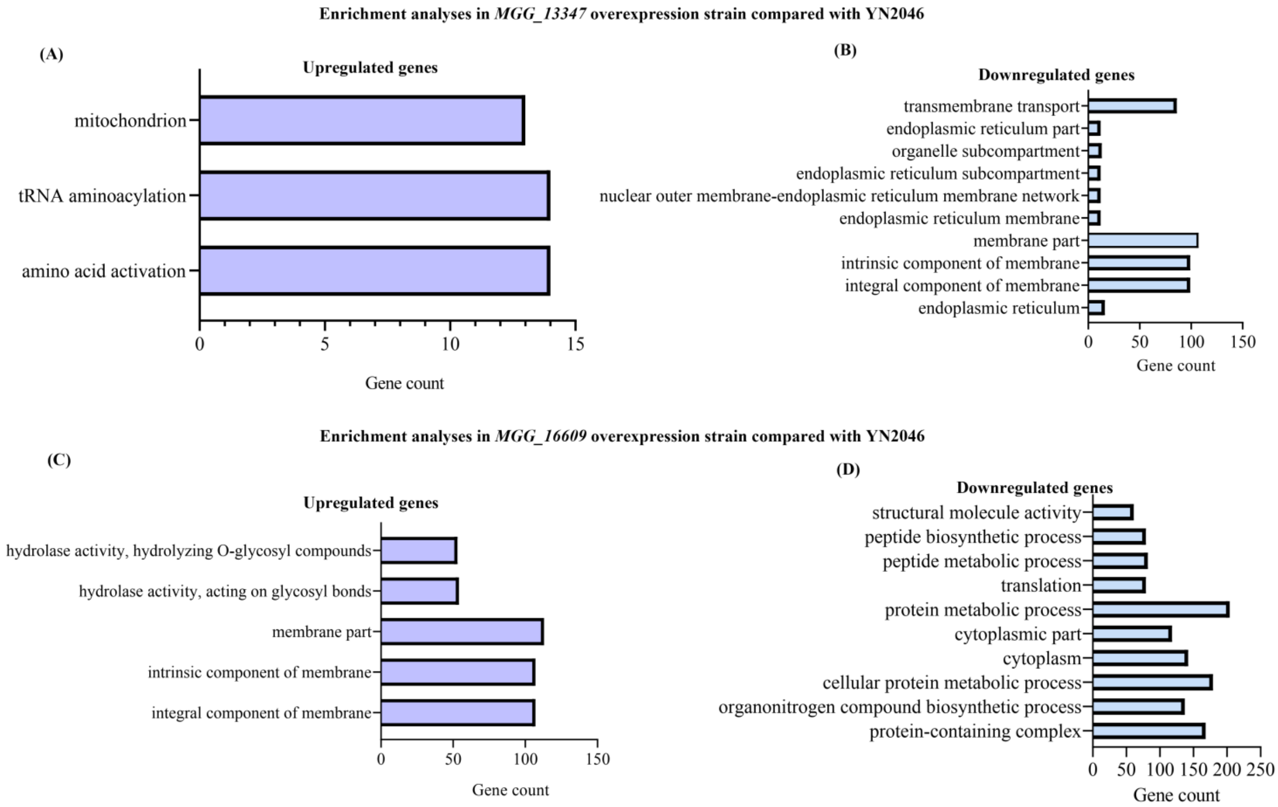

Figure S8. The enrichment analysis of DEGs in *MGG_13347* and *MGG_16609* overexpression strains compared with that in YN125 under normal condition. The GO enrichment analyses for upregulated (A) and downregulated (B) genes in *MGG_13347* overexpression strains under normal condition. The GO enrichment analyses for upregulated (C) and downregulated (D) genes in *MGG_16609* overexpression strains under normal condition. The top 10 GO terms with the highest *p*-value are present in the panel B and D.

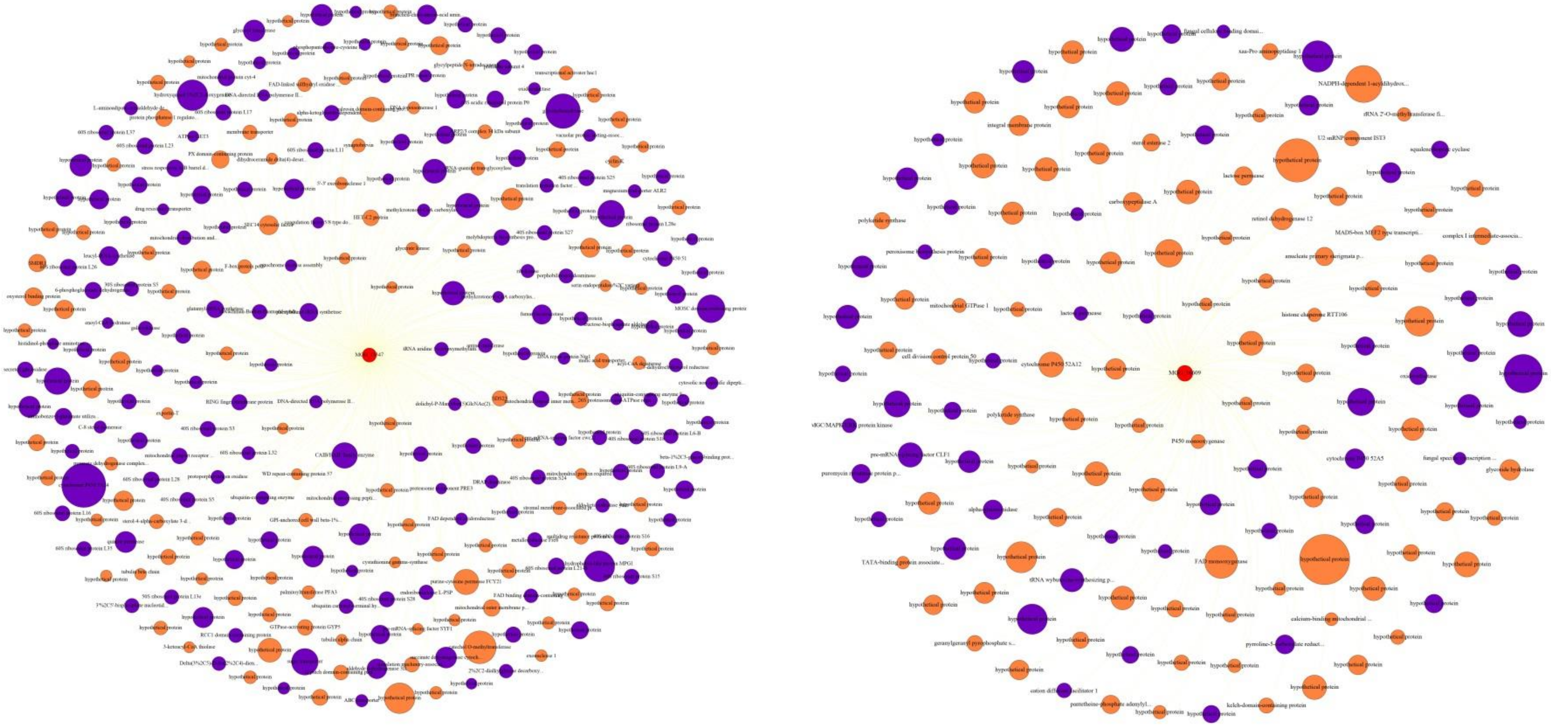

MGG_13347 ***MGG_16609***

Figure S9. The interacting gene networks of *MGG_13347* (left) and *MGG_16609* (right) in respond to excessive Mn^{2+}. The nodes with green or red means the downregulated or upregulated genes in *MGG_13347* and *MGG_16609* overexpression strains, respectively. The size of nodes represents the absolute value of fold change.
